# Size Effect of a Piezoelectric Patch on a Rectangular Plate with the Neural Network Model

**DOI:** 10.3390/ma14123240

**Published:** 2021-06-11

**Authors:** Hequn Min, Jie Zhang, Mu Fan

**Affiliations:** 1 Key Laboratory of Urban and Architectural Heritage Conservation, Ministry of Education, School of Architecture, Southeast University, Nanjing 210096, China; hqmin@seu.edu.cn; 2 Interdisciplinary Research Institute of Aeronautics and Astronautics, College of Aerospace Engineering, Nanjing University of Aeronautics and Astronautics, Nanjing 210010, China; nuaa_zhangjie@nuaa.edu.cn

**Keywords:** piezoelectric effect, neural network, active actuating, rectangular plate, size effect

## Abstract

Artificial neural networks have been widely used in many studies, such as the prediction of the piezoelectric effect of the plate of engineering structures in vibration and noise reduction. In this paper, an artificial neural network (ANN) model was employed to explore the piezoelectric patch size and thickness’s effect on the first order natural frequency and displacement amplitude of a plate. With the finite element method (FEM), a rectangular plate actuated by a piezoelectric patch was analyzed with various patch sizes. The FEM data was later used to build an ANN model. The dynamic response of the plate was predicted by the ANN model and validated with FEM in terms of 1st order natural frequency and displacement amplitude. Results from case studies showed that with the input of patch length, width and thickness, ANN model can accurately predict both natural frequency and displacement amplitude. When the input of ANN model was simplified to patch size and thickness or the volume of the patch, the accuracy became worse and worse. The influence of the patch size and thickness on the first order natural frequency was coupled and the maximal and minimal values were predicted based on the ANN model.

## 1. Introduction

In the past few decades, piezoelectric devices triggered or driven by a mechanical deformation have been invented and used in various engineering systems and applications, such as transducers, electromechanical sensors, actuators, and energy generators. Since the piezoelectric phenomenon was first observed in the 1880s [1], later generations proposed and perfected the complex piezoelectric theory. The piezoelectric effect includes the direct piezoelectric effect and the converse piezoelectric effect. Generally, the direct piezoelectric effect is that the piezoelectric material responds to mechanical force/pressure and generates an electric charge/voltage. On the contrary, the electric charge/field applied to the material through the converse piezoelectric effect can cause mechanical stress or strain. For the direct piezoelectric effects which are commonly used in engineering sensing and energy harvesting, Krommer et al. studied the quasi-static thermal bending and free vibration, forced vibration, and driving vibration of polygonal plates with simply-supported edges [2]. Tzou et al. first formulated a piezoelectric finite element with higher internal degrees of freedom and derived the structure identification and control using piezoelectric finite element, and finally presented the state variable conversion of the dynamic equation [3]. Zhang et al. investigated the topology optimization of the electrode coverage over piezoelectric patches attached to a thin-shell structure, in order to reduce the energy consumption of active vibration control under harmonic excitations [4]. The converse piezoelectric effect can be applied to structure control and active actuating. Zhang and Kang’s paper investigates topology optimization of the piezoelectric actuator/sensor coverage attached to a thin-shell structure to improve the active control performance for reducing the dynamic response under transient excitations. The proposed method can be used for providing useful guidance to the layout design of the actuator/sensor layers attached to a thin-shell structure subject [5]. Tzou et al. used a distributed actuator layer to suppress the oscillation caused by the converse piezoelectric effect and demonstrated the application of this structure in distributed dynamic measurement and control [6]. Shi et al. investigated the response of a piezoelectric cantilever to an applied external voltage [7]. Tzou et al. proposed and evaluated an active adaptive structure made of piezoelectric materials and studied the frequency manipulation of the piezoelectric bimorph beam theoretically and experimentally [8]. Reddy et al. presented a simple negative speed feedback control algorithm that combines the direct and the converse piezoelectric effects to actively control the dynamic response of the integrated structure through closed-loop control [9]. All these studies have shown that the field of piezoelectric effect research has experienced tremendous growth in research and development.

Artificial neural networks are a research hotspot since it emerged in the field of artificial intelligence back in the 1980s. The research of artificial neural networks can be traced back to the perceptron model proposed by Rosenblatt in 1957. With the continuous deepening of research work in the past ten years, artificial neural networks have made considerable progress. Successfully solved many problems in the fields of pattern recognition [10,11], intelligent robots [12], automatic control [13], predictive estimation [14,15], biology [16,17,18], medicine [19,20,21], and economics [22,23]. In the field of using artificial neural networks to achieve prediction, some documents mention the application of neural network prediction in practical engineering problems, vibration control of mechanical systems, medicine, and other fields. Khandelwal et al. tried to evaluate and predict the ground vibration and frequency caused by the explosion by using artificial neural network (ANN) technology to combine rock properties, explosion design, and explosion parameters and successfully trained a model with fifteen hidden neurons and ten inputs three-layer feedforward backpropagation neural network with parameters and two output parameters [24]. Gebraeel et al. developed a neural network-based model to predict bearing failures. In the best model, 62% of the predicted value has an error of less than 10% from the actual value. When the error is increased to 20%, the ratio is 92% [25]. Through a case study, Maier et al. used neural network methods to predict the salinity of the Murray River (South Australia) 14 days in advance. Using real-time data in 1991, the average absolute percentage error obtained was 6.5% [26]. Papadrakakis et al. use evolutionary strategies and neural networks for structural optimization. The trained neural network is used to predict, within an acceptable accuracy, the values of the objective and constraint functions. The numerical tests presented to demonstrate the computational advantages of the proposed approach which become more pronounced in large-scale optimization problems [27]. Feng et al. proposed a novel approach for the prediction of residual useful life (RUL) of structures through appropriately combining the phase-field method and neural network. The proposed approach is a hybrid model of both physical and data-driven techniques, which can build a bridge between traditional computational fracture mechanics and deep learning algorithms [28].

The study presented in this paper introduces an artificial neural network model which is the most widely used multi-layer feedforward neural network trained according to the error backpropagation algorithm to predict the vibration and natural frequency of a piezoelectric rectangular plate. The purpose of this study was to examine the key parameters that may influence the vibration amplitude and natural frequency of a piezoelectric rectangular plate. The first natural frequency of the piezoelectric rectangular plate and the displacement amplitude of the center position of the upper surface of the piezoelectric layer are discussed with respect to varying the side length and thickness of the piezoelectric patch. Comparisons between the finite element results and the artificial neural network (ANN) predicted results were made to verify the effectiveness of the method.

## 2. Physical Model

### 2.1. Dynamic Equation of the Thin Plate

In the physical model, a flexible thin plate with four edges clamped conditions was adopted. The governing equation of the plate can be formulated as a partial differential equation (PDE) together with the corresponding boundary conditions and by assuming small lateral deflection conditions, one may have [29]:

(1) $$\frac{\partial^{4}w\left(x,y,t\right)}{\partial x^{4}}+2\frac{\partial^{4}w\left(x,y,t\right)}{\partial x^{2}\partial y^{2}}+\frac{\partial^{4}w\left(x,y,t\right)}{\partial y^{4}}+\frac{\rho h}{D}\frac{\partial^{2}w\left(x,y,t\right)}{\partial t^{2}}=\frac{q\left(x,y,t\right)}{D}$$

where $w\left(x,y,t\right)$ denotes the deflection of the mid-plane of the plate, ρ is the density of plate with dimension mass per unit volume, q is the transverse external force with the dimension of force per unit area, $D=\left(Eh^{3}\right)/\left(12\left(1-\upsilon^{2}\right)\right)$ is the flexural rigidity, E is the elastic modulus of the plate material, h is the thickness and υ is the Poisson’s ratio. In the rectangular coordinate system, for x=a, y=b are clamped edges, then the boundary condition is as follows:

(2) $$\left\{\begin{array}{c} {\left.w\right|}_{x=a}=\frac{\partial w}{\partial x}=0\\ {\left.w\right|}_{y=b}=\frac{\partial w}{\partial y}=0 \end{array}\right.$$

For simulation purposes, it is natural to assume that the forces and moments of the plate due to its weight are neglected. In other words, the plate has no deflection initially. Thus, for every point located on the plate, the displacement at t=0 is assumed to be zero:

(3) $${\left.W\right|}_{t=0}=0$$

On the other hand, in the case of a plane stress problem, the constitutive equation of a homogeneous isotropic piezoelectric element can analogy for the one-dimensional problem studied by Lee and Kim [30] and Park [31], written as:

(4) $$\left[\begin{array}{c} \sigma_{11}\\ \sigma_{22}\\ 2\sigma_{12}\\ {\bar{E}}_{3} \end{array}\right]=\left[\begin{array}{cccc} \frac{E_{p}^{E}}{1-v_{p}^{2}} & \frac{E_{p}^{E}v_{p}}{1-v_{p}^{2}} & 0 & -h_{31}\\ \frac{E_{p}^{E}v_{p}}{1-v_{p}^{2}} & \frac{E_{p}^{E}}{1-v_{p}^{2}} & 0 & -h_{31}\\ 0 & 0 & G_{p}^{E} & 0\\ -h_{31} & -h_{31} & 0 & \beta_{33}^{T} \end{array}\right]\left[\begin{array}{c} \varepsilon_{11}\\ \varepsilon_{22}\\ 2\varepsilon_{12}\\ {\bar{D}}_{3} \end{array}\right]$$

where $\sigma_{ij}$ and $\varepsilon_{ij}$ are the stress and strain components, respectively. ${\bar{E}}_{i}$ and ${\bar{D}}_{i}$ denote the components of the electrical field and electrical displacement, correspondingly. The superscripts (E) and (T) indicate that the material properties were measured under constant electrical and tension fields, respectively. Moreover, $h_{31}$ is the piezoelectric constant, $\beta_{33}^{T}$ interprets the dielectric constant measured under a constant tension field, and $v_{p}$ denotes the appropriate Poisson’s ratio. Equation (4) indicates the existence of two equal in-plane strains $\varepsilon_{11}$ and $\varepsilon_{22}$ for any applied voltages along the polling axis of the piezoelectric patch. The piezoelectric patch thickness is presumably small enough to maintain a uniform and constant electric displacement over its thickness.

**Figure 1:**
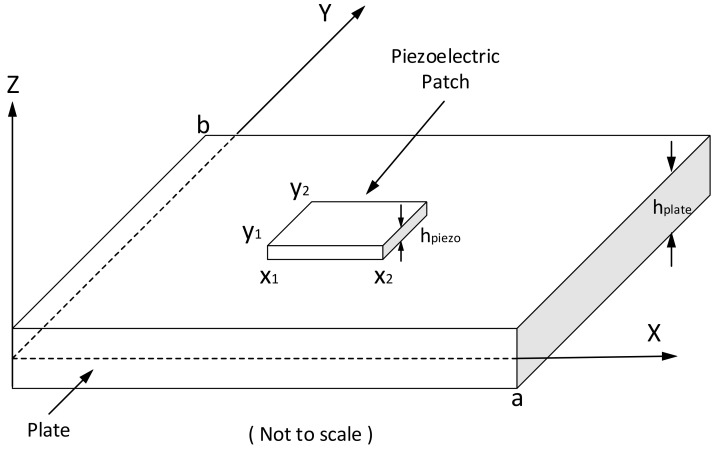
The dynamic system of the thin plate.

As shown in Figure 1, consider bonding a piezoelectric patch on the upper surface of a thin rectangular plate with the *Z*-axis pointing upward. It is assumed that the piezoelectric patch layer covers the plate from locations $x_{1}$ to $x_{2}$ in the X direction and from $y_{1}$ to $y_{2}$ in the Y direction. The dimensions of the piezo patch along the X and Y axes are specified by $a_{s}$ and $b_{s}$, respectively. The kinetic energy of the plate and the piezo patch are the following:

(5) $$K=K_{plate\;}+K_{piezo}$$

where:

(6) $$K_{plate\;}=\frac{1}{2}\int_{A}\mu_{plate\;}{\dot{w}}^{2}dA$$

and:

(7) $$K_{piezo\;}=\frac{1}{2}\int_{A}\mu_{piezo\;}{\dot{w}}^{2}\Delta H\left(x,y\right)dA$$

In the above equations, μ represents the mass per unit area, A represents the area of the plate, and $\left(\cdot \right)$ represents the derivatives concerning time. Also:

(8) $$\Delta H\left(x,y\right)=\left[H\left(x-x_{1}\right)-H\left(x-x_{2}\right)\right]\left[H\left(y-y_{1}\right)-H\left(y-y_{2}\right)\right]$$

where H is the Heaviside step function. On the other hand, assuming that the strain is extremely small, the strain energy of the plate and the piezoelectric patch becomes:

(9) $$U=U_{plate\;}+U_{piezo\;}$$

where:

(10) $$U_{\mathrm{plate}\;}=\frac{1}{2}\int_{A}D_{\mathrm{plate}\;}\left[w_{xx}^{2}+w_{yy}^{2}+2vw_{xx}w_{yy}+2\left(1-v\right)w_{xy}^{2}\right]dA$$

and:

(11) $$\begin{array}{cc} U_{piezo\;} & =\frac{1}{2}\int_{V}\left[\varepsilon^{T}\sigma +\bar{ED}\right]dV\\ & =\frac{1}{2}\int_{A}\left\{D_{piezo\;}\left[w_{xx}^{2}+w_{yy}^{2}+2v_{p}w_{xx}w_{yy}+2\left(1-v_{p}\right)w_{xy}^{2}\right]\right.\\ & +\left[h_{31}h_{piezo\;}{\bar{D}}_{3}\left(h_{piezo\;}+h_{plate\;}\right)\right]\left[w_{xx}+w_{yy}\right]\\ & \left.+h_{piezo\;}\beta_{33}^{T}{\bar{D}}_{3}^{2}\right\}\Delta H\left(x,y\right)dA \end{array}$$

The parameter $D_{\mathrm{piezo}\;}=\frac{E_{p}^{E}\left[4h_{\mathrm{piezo}\;}^{3}+3h_{\mathrm{plate}\;}h_{\mathrm{piezo}\;}\left(h_{\mathrm{plate}\;}+2h_{\mathrm{piezo}\;}\right)\right]}{12\left(1-v_{p}^{2}\right)}$ is the bending stiffness of the piezoelectric patch, $h_{\mathrm{piezo}\;}$ is the thickness of the piezo patch and $z=h_{\mathrm{piezo}\;}\left(h_{\mathrm{plate}\;}+2h_{\mathrm{piezo}\;}\right)$. Finally, applying Hamilton’s principle:

(12) $$\delta H=\int_{t1}^{\mathrm{t2}}\left(\delta U-\delta K\right)dt=0$$

Thus, the following constitutive equations of system motion and related natural and geometric boundary conditions are derived [32]:

(13) $$\begin{array}{c} D_{plate\;}\nabla^{4}w+\mu_{plate\;}\frac{\partial^{2}w}{\partial t^{2}}+D_{piezo\;}\left\{\left[\nabla^{4}w+\left(\frac{\mu_{piezo\;}}{D_{piezo\;}}\right)\frac{\partial^{2}w}{\partial t^{2}}\right]\Delta H\left(x,y\right)\right.\\ +2\left[w_{xxx}+w_{xyy}\right]\Delta H_{1,x}\left(x,y\right)+2\left[w_{yyy}+w_{xxy}\right]\Delta H_{1,y}\left(x,y\right)\\ +2\left[\left(1-v_{p}\right)w_{xy}\right]\Delta H_{1,xy}+\left(1+v_{p}\right)w_{xx}\Delta H_{1,xx}\left(x,y\right)\\ +\left(1+v_{p}\right)w_{yy}\Delta H_{1,yy}\left(x,y\right)=f\left(x,y,t\right)\\ -\frac{1}{2}\left[h_{31}{\bar{D}}_{3}z\nabla^{2}\Delta H\left(x,y\right)\right] \end{array}$$

where the term $\nabla^{4}$ is a differential operator equivalent to $\frac{\partial^{4}}{\partial x^{4}}+2\frac{\partial^{2}}{\partial x\partial y}+\frac{\partial^{4}}{\partial y^{4}}$, and $w_{xyy}$ stands for finding the partial derivative of x first and then finding the partial derivative of y twice.

To solve Equation (13), one needs to assume that the thickness ratio of the piezoelectric plate is small so that the position of the neutral plane of the main plate can be kept unchanged with acceptable accuracy in the presence of the piezoelectric patch. Besides, since in most practical situations, the elastic modulus of piezoelectric materials is smaller than that of the motherboard, the position of the neutral plane of the combined system relative to the motherboard changes even less. In the open literature, the effect of piezoelectric patch dimension on the dynamic responses was usually ignored so that Equation (13) can be simplified to a pure elastic plate model. However, when the piezoelectric patch size is comparable to the plate, the dimension of the piezoelectric patch can significantly influence the natural frequency and displacement amplitude of the structure, which may cause inaccuracy to vibration and sounds radiation control. In the next parts, an ANN model will be built with the help of finite element simulation to explore the dimension effect of the piezoelectric patch on the structural dynamic responses.

### 2.2. Neural Network Modeling

The main advantage of ANN is that it can model a problem using examples rather than analytical descriptions. To study the dynamic behavior of the rectangular thin plate system considering the piezoelectric patch size effect, a computer program was written to conduct in-depth research with the help of the neural network toolbox. The complete algorithm structure of a conventional ANN includes at least three different layers: the input layer, hidden layer, and output layer. Each neuron inter-connects with all the neurons in the following layer. With a proper activation function, a combination of optimized weights can generate the prediction of the dependent variable:

(14) $$NET=\sum_{i,j}^{n}w_{ij}x_{i}+c$$

where $w_{ij}$ represents the weight value of a connection, $x_{i}$ represents an inputted independent variable, and c represents a deviation. For the activation function, the logsig function in Equation (15) is the sigmoid function in logistic regression. The input value of the Log-sigmoid function can take any value, and the output value is between 0 and 1. The input and output values of the linear transfer function purelin can take any value:

(15) $$log\;sig\left(NET\right)=\frac{1}{1+e^{-NET}}$$

An ANN model needs to be trained from an existing training set including many pairs of input-output elements., until the root mean square error (*RMSE*) between the training output data and the predicted output are minimized, as given in Equation (16):

$$RMSE=\sqrt{\frac{\sum_{i}^{n}{\left(P_{i}-A_{i}\right)}^{2}}{n}}$$

where $P_{i}$ represents the predicted value outputted by the ANN, $A_{i}$ is the actual value, and n represents the total number of samples.

**Figure 2:**
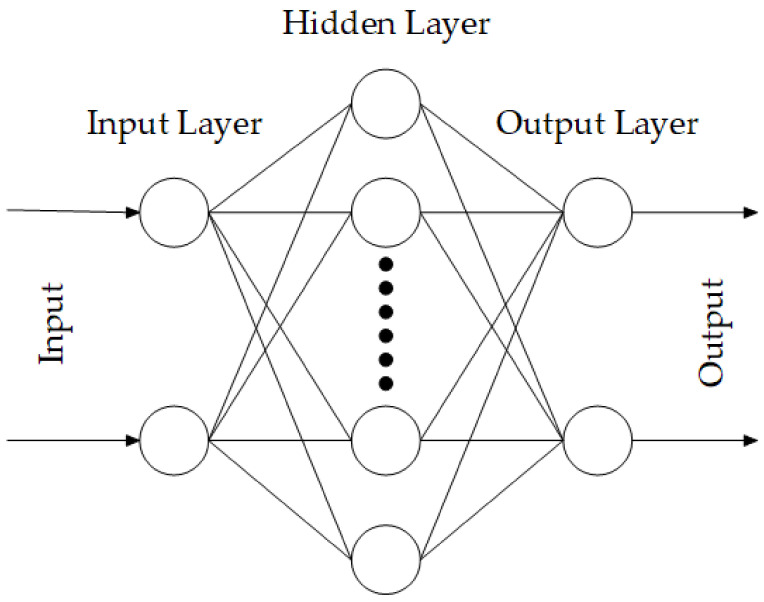
Schematic of the neural network structure.

In this study, a typical multilayer neural network with one hidden layer is used, the network has two inputs and two output values. The proposed ANN model shown in Figure 2 was trained by the input data obtained from COMSOL. The input layer needs many sets of data including the side length of the plate and the thickness of the piezoelectric patch. Also, the output layer gives the predicted natural frequency and displacement amplitude. The neurons in hidden layers all use a sigmoid transfer function and the neurons in the output layer use a linear transfer function. Trainbr (Bayesian regularization algorithm) modified the Levenberg-Marquardt algorithm to improve the generalization ability of the network. At the same time, the difficulty of determining the optimal network structure is reduced. The interconnection between processing units in a neural network is distributed through weights. These weights can be adjusted through the training process to optimize the neural network output.

## 3. Case Study

In this section, an example of a rectangular plate with four clamped edges and with a single rectangular piezoelectric patch on the upper surface is used to verify the model introduced in this study and demonstrate the neural network-based prediction method. Lower mode vibration usually dominates the dynamic responses of beams and plates. Hence, we studied the first order natural frequency and the corresponding displacement (at the center point of the plate). In case 1, the side length and thickness of the square piezoelectric patch are selected as the two inputs of the neural network, and the accuracy of the model is verified by comparing the prediction result of the neural network model with the calculation result of the finite element software. In case 2, the area and thickness of the rectangular piezoelectric patch are selected as inputs, and the influence of the same area with different aspect ratios on the prediction results is analyzed. In case 3, the volume of the rectangular piezoelectric patch is used as the only input, and the difference between the other two neural network models is compared.

### 3.1. CASE 1: Input Side Length and Thickness

Create a three-dimensional model in the COMSOL software, keep the size and thickness of the aluminum plate fixed, and attach the square piezoelectric patch to the center of the surface of the square aluminum plate. To implement the ANN prediction, this study considered a 1 mm thick aluminum plate with the parameters given in Table 1 and all edges clamped. The dimensions of the plate are a = 100 mm, b = 100 mm, and $h_{plate}$ = 1 mm. The density, modulus of elasticity, and Poisson’s ratio are ρ = 2700 $\mathrm{kg}/m^{3}$, E = 70 GPa, υ = 0.33, respectively. In most cases, damping has little effect on the mode shape and characteristic frequency. However, when the excitation frequency is near the natural frequency (for example, within ±50%), the damping model is critical. When approaching the resonance frequency, the result is completely controlled by damping. So, for all vibration modes, the damping loss coefficient was assumed as *ζ* = 0.01. The size of the square piezoelectric plate is from 1 mm × 1 mm to 100 mm × 100 mm, and the thickness of $h_{piezo}$ is from 1.0 mm to 2.0 mm. As shown in Table 2, the material properties are $\rho_{piezo}$ = 7500 $\mathrm{kg}/m^{3}$, $E_{p}$ = 56 GPa, $\upsilon_{p}$ = 0.36, respectively.

**Figure 3:**
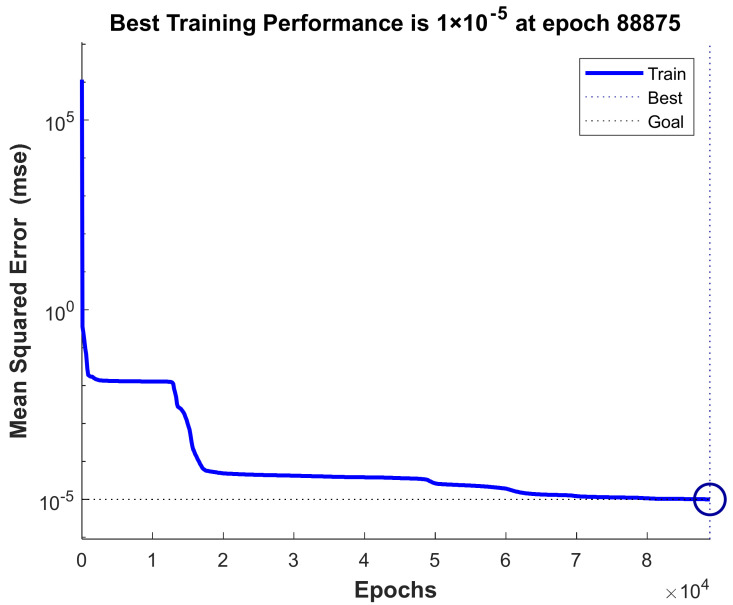
Performance of the proposed ANN model.

Code was written to use the artificial neural network toolbox to train the simulation data obtained from COMSOL. Among all the 658 sets of simulation data, 398 sets of data are selected as the training samples of the artificial neural network, and the rest are used to test the effect of the artificial neural network after training. As can be seen from Figure 3, the performance results of the proposed 2-72-2 ANN model, which shows that the mean square error of the network is initially large, and then decreases to a smaller value as the number of learning increases. This means the network was learning and one epoch is equivalent to training once using all samples in the training set. Training on the training vectors continued as long as the training reduced the network’s error on the validation vectors. Eventually, meet the expected accuracy requirements.

**Figure 4:**
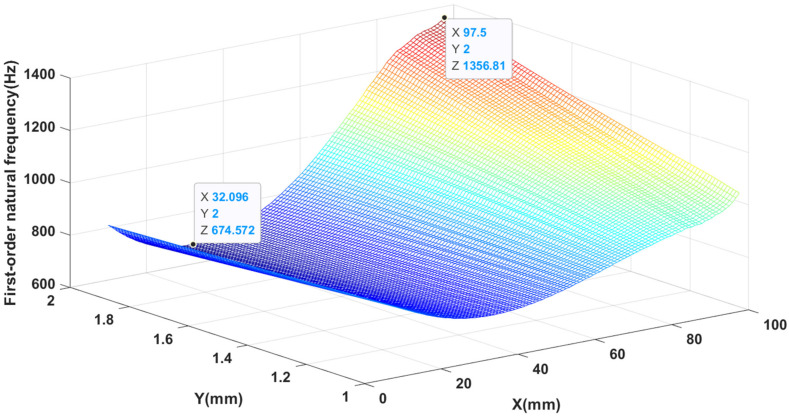
The variation of first-order natural frequency with side length and thickness.

**Figure 5:**
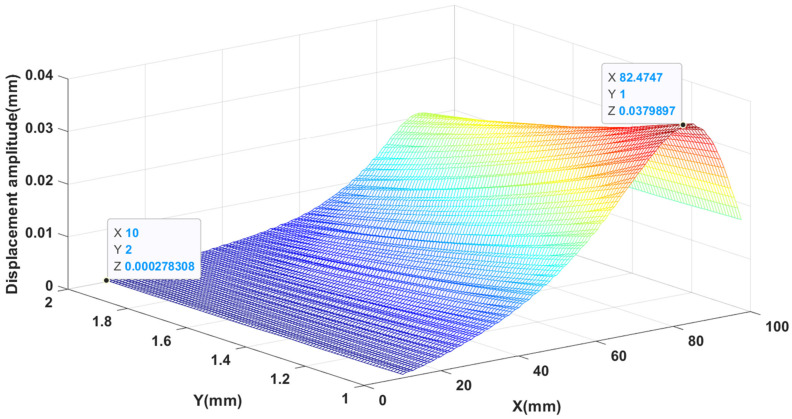
Displacement amplitude varies with side length and thickness.

The results of the finite element simulation and the results of the proposed ANN model were compared in Table 3. The accuracy of the ANN prediction is good. The influence of patch dimensions on the first order natural frequency and displacement amplitude is shown in Figure 4 and Figure 5, respectively. It can be seen from Figure 4 that when the thickness of the piezoelectric patch is constant, the first-order natural frequency first decreases and then increases as the side length of the piezoelectric patch increases. When keeping the side length of the piezoelectric patch constant, when the side length of the piezoelectric patch is small (approximately less than 57.7 mm), the first-order natural frequency decreases with the increase of thickness, and when the side length is larger (approximately greater than 57.7 mm), the first-order natural frequency increases as the thickness increases. It is easy to see that when the side length is equal to 97.5 mm and the thickness is 2.0 mm, the first-order natural frequency reaches its maximum value, which is 1356.81 Hz. When the side length is equal to 32.1 mm and the thickness is 2.0 mm, the minimum value is reached, which is 674.57 Hz.

As shown in Figure 5, when the thickness of the piezoelectric patch remains unchanged, the displacement amplitude first increases and then decreases as the side length increases. When the side length of the piezoelectric patch remains unchanged, the displacement amplitude decreases as the thickness increases. Obviously, when the side length is 82.5 mm and the thickness is 1.0 mm, the displacement amplitude is 37.99 × $10^{-3}$ mm, which is the maximum. When the side length is 10 mm and the thickness is 2.0 mm, the displacement amplitude is 0.28 ×$\;10^{-3}$ mm, which is the minimum.

### 3.2. CASE 2: Input Area and Thickness

**Figure 6:**
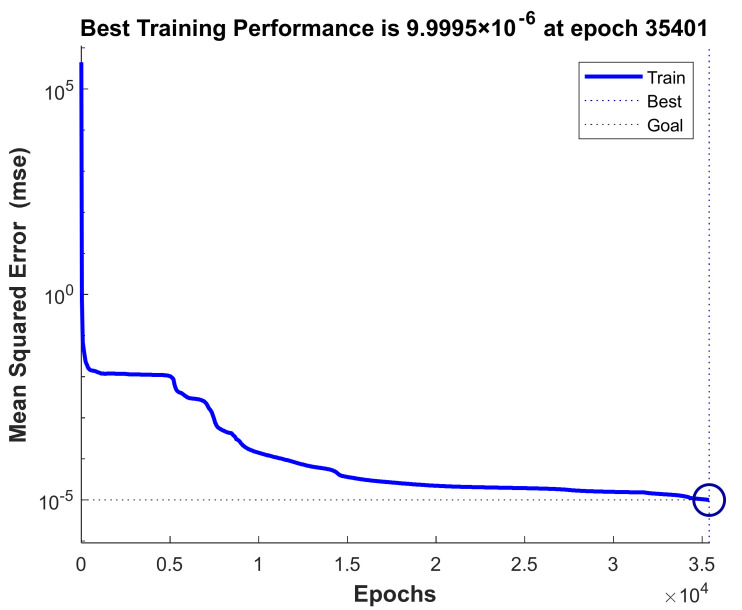
Performance of the proposed ANN model.

Based on the case study of the previous neural network model, the input of the model was adjusted, the area and thickness of the piezoelectric patch were used as the input, and the output was still the first-order natural frequency and the displacement amplitude remained unchanged. It can be seen from Figure 6 that the mean square error of the adjusted 2-71-2 ANN model after training meets the expected requirements.

As before, we also used COMSOL to calculate the corresponding data to compare with the prediction results of the ANN model. From Table 4, we can find that the adjusted ANN model maintains good accuracy. Not only that, but we also used finite element software to calculate the data of 132 groups of rectangular piezoelectric patches with the same area but different aspect ratios. As shown in Table 5, comparing them with the data predicted by the neural network, it is found that as the aspect ratio of the piezoelectric patch increases, the error of the prediction also increases. In other words, when the area and thickness of the piezoelectric patch are the same, the change of the aspect ratio will also cause the change of the first-order natural frequency and the displacement amplitude.

**Figure 7:**
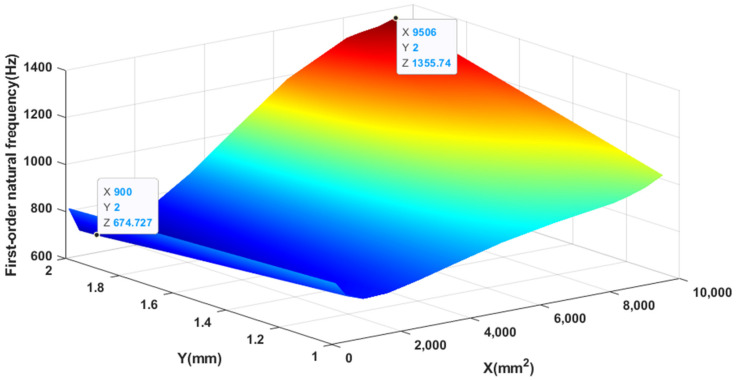
The variation of first-order natural frequency with area and thickness.

Figure 7 shows the relationship between the area, thickness, and first-order natural frequency of the piezoelectric patch. When keeping the thickness of the piezoelectric patch constant, as the area of the piezoelectric patch increases, the first-order natural frequency first decreases and then increases. When the area of the piezoelectric patch is less than 3408 mm^2^ and remains unchanged, the first-order natural frequency decreases as the thickness of the piezoelectric patch increases; on the contrary, when the area is greater than 3408 mm^2^ and remains unchanged, the first-order natural frequency increases with As the thickness of the piezoelectric patch increases, it increases. The maximum and minimum values are marked in the figure, that is when the area is equal to 9506 mm^2^ and the thickness is 2.0 mm, the first-order natural frequency reaches the maximum value, which is 1355.74 Hz. When the area is equal to 900 mm^2^ and the thickness is 2.0 mm, the minimum value is 674.73 Hz.

**Figure 8:**
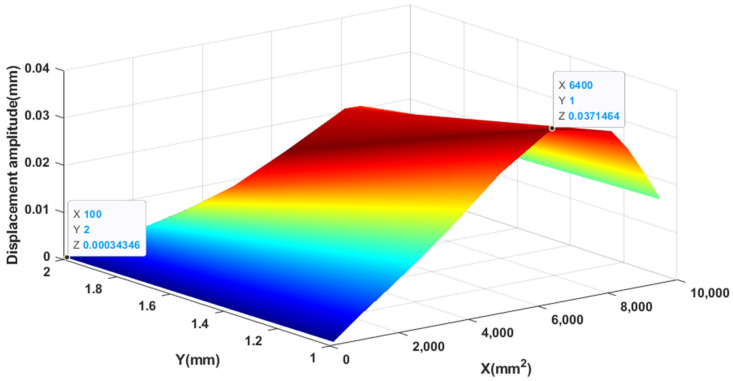
Displacement amplitude varies with area and thickness.

As shown in Figure 8, when the thickness of the piezoelectric patch remains unchanged, the displacement amplitude shows a trend of first increasing and then decreasing with the increase of the area of the piezoelectric patch. When the area of the piezoelectric patch remains the same, the displacement amplitude decreases as the thickness increases. Obviously, when *X* is 6400 mm^2^ and Y is 1.0 mm, the maximum is 37.15 × $10^{-3}$ mm; when *X* is 100 mm^2^ and Y is 2.0 mm, the minimum is 0.34 × $10^{-3}$ mm.

### 3.3. CASE 3: Input Volume

In this part, the input of the ANN model is reduced, and only the volume of the piezoelectric patch is input as a known quantity, but the output remains the same, that is, the first-order natural frequency and displacement amplitude. Through this single-input and two-output ANN model, the effectiveness of the model introduced in this article is verified, and it is compared with the previous two cases to analyze the impact of changing the input on the accuracy of the ANN model.

It can be seen from Table 6 that the results of the ANN model with only a single input after training are not particularly accurate. Compared with the previous two cases, the prediction error is much larger, whether it is for the first order natural frequency or the displacement amplitude. This was because the change of the patch shape/volume could affect the distribution of piezoelectric induced control force and moment. However, the prediction results of first order natural frequency were in good accuracy in cases 2 and 3, which made the two models also valuable. 

## 4. Discussion

In this study, an artificial neural network model was employed to explore the size effect of piezoelectric patches on a rectangular plate. Taking a rectangular plate with four sides clamped as our case study, the dynamic response of the plate was predicted by the ANN model and validated with FEM in terms of first order natural frequency and displacement amplitude. With thorough analyses of various parameters, it was found that with the input of patch length, width, and thickness, the ANN model can accurately predict both natural frequency and displacement amplitude; when the input of the ANN model was simplified to patch size and thickness or the volume of the patch, the accuracy became worse and worse. When the area and thickness of the piezoelectric patch are the same, the change of the aspect ratio will also cause the change of the first order natural frequency and the displacement amplitude. The influence of the patch size and thickness on the first order natural frequency was coupled and the maximal and minimal values were predicted based on the ANN model.

## 5. Conclusions

An artificial neural network (ANN) model was proposed to quickly and effectively predict the displacement amplitude and natural frequency of the piezoelectric actuated rectangular plate. The original data was obtained with COMSOL software with varying piezoelectric patch sizes and thickness. With the ANN model, the relationship between the first order natural frequency, the displacement amplitude, and the key parameters including the side length, thickness, area, and volume of the piezoelectric patch were discussed. The prediction results and errors based on the ANN model were demonstrated and proved to be acceptable. The work in this study provides a convenient and effective method to predict the dynamic responses of the piezoelectric actuated plate with considering piezoelectric patch dimension effect. It is not only suitable for four sides clamped but also for other more complicated boundary conditions.

## Figures and Tables

**Table 1:** Plate specifications.

Parameter	Value
Length a, mm	100
Width b, mm	100
Thickness $h_{plate\;}$, mm	1.0
Density ρ, $\mathrm{kg}/m^{3}$	2700
Modulus of elasticity E, Pa	70 ×$\;10^{9}$
Poisson ratio υ	0.33

**Table 2:** The piezoelectric patch properties.

Parameter	Value
Length a, mm	1.0~100
Width b, mm	1.0~100
Thickness $h_{piezo}$, mm	1.0~2.0
Density $\rho_{piezo}$, $\mathrm{kg}/m^{3}$	2500
Modulus of elasticity $E_{p}$, Pa	56 × $10^{9}$
Poisson ratio $\upsilon_{p}$	0.36

**Table 3:** Training and predicted results of 2-72-2 ANN model.

Length	Thickness/mm	First-Order Natural Frequency/Hz		Error/%	Displacement Amplitude/mm		Error/%
		COMSOL	ANN		COMSOL	ANN	
10.0	1.0	856.87	856.87	0.00	0.69 × $10^{-3}$	0.72 × $10^{-3}$	4.35
15.0	1.1	816.18	816.18	0.00	1.38 × $10^{-3}$	1.32 × $10^{-3}$	−4.35
20.0	1.2	778.09	778.10	0.00	2.11 × $10^{-3}$	2.09 × $10^{-3}$	−0.95
22.5	1.3	757.29	757.29	0.00	2.33 × $10^{-3}$	2.35 × $10^{-3}$	0.86
27.5	1.4	733.13	733.13	0.00	2.91 × $10^{-3}$	2.90 × $10^{-3}$	−0.34
35.0	1.5	722.68	722.68	0.00	3.82 × $10^{-3}$	3.83 × $10^{-3}$	0.26
40.0	1.6	725.77	725.77	0.00	4.20 × $10^{-3}$	4.21 × $10^{-3}$	0.24
57.5	1.7	841.62	841.62	0.00	7.18 × $10^{-3}$	7.13 × $10^{-3}$	−0.70
65.0	1.8	930.04	930.04	0.00	8.63 × $10^{-3}$	8.61 × $10^{-3}$	−0.23
82.5	1.9	1202.42	1202.42	0.00	17.46 × $10^{-3}$	17.45 × $10^{-3}$	−0.06
97.5	2.0	1355.75	1356.81	0.08	15.87 × $10^{-3}$	15.69 × $10^{-3}$	−1.13

**Table 4:** Training and predicted results of 2-71-2 ANN model.

Area	Thickness/mm	First-Order Natural Frequency/Hz		Error/%	Displacement Amplitude/mm		Error/%
		COMSOL	ANN		COMSOL	ANN	
100	1.0	856.87	856.87	0.00	0.69 × $10^{-3}$	0.83 × $10^{-3}$	20.23
400	1.1	787.51	787.51	0.00	2.38 × $10^{-3}$	2.29 × $10^{-3}$	−3.74
900	1.2	750.04	750.05	0.00	4.27 × $10^{-3}$	4.28 × $10^{-3}$	0.13
1600	1.3	751.44	751.44	0.00	6.07 × $10^{-3}$	6.10 × $10^{-3}$	0.47
2500	1.4	791.77	791.77	0.00	7.94 × $10^{-3}$	7.91 × $10^{-3}$	−0.39
3600	1.5	867.70	867.70	0.00	10.29 × $10^{-3}$	10.29 × $10^{-3}$	−0.05
4900	1.6	982.22	982.22	0.00	13.87 × $10^{-3}$	13.74 × $10^{-3}$	−0.93
6400	1.7	1117.52	1117.52	0.00	18.99 × $10^{-3}$	18.98 × $10^{-3}$	−0.07
8100	1.8	1238.61	1238.61	0.00	23.26 × $10^{-3}$	23.22 × $10^{-3}$	−0.16

**Table 5:** Comparison of prediction results of the same area with different aspect ratios.

Area	Thickness/mm	First-Order Natural Frequency/Hz		Error/%	Displacement Amplitude/mm		Error/%
		COMSOL	ANN		COMSOL	ANN	
100	1.0	856.87	856.87	0.00	0.69 × $10^{-3}$	0.83 × $10^{-3}$	20.23
4 × 25		869.34		−1.43	1.06 × $10^{-3}$		−21.39
2.5 × 40		878.65		−2.48	1.34 × $10^{-3}$		−38.20
2 × 50		881.17		−2.76	1.37 × $10^{-3}$		−39.56
400	1.1	787.51	787.51	0.00	2.38 × $10^{-3}$	2.29 × $10^{-3}$	−3.74
16 × 25		790.67		−0.40	2.46 × $10^{-3}$		−7.17
10 × 40		816.50		−3.55	3.23 × $10^{-3}$		−29.18
5 × 80		851.29		−7.49	3.52 × $10^{-3}$		−35.04
900	1.2	750.04	750.05	0.00	4.27 × $10^{-3}$	4.28 × $10^{-3}$	0.13
25 × 36		754.72		−0.62	4.42 × $10^{-3}$		−3.27
18 × 50		783.56		−4.28	5.52 × $10^{-3}$		−22.48
15 × 60		802.80		−6.57	6.52 × $10^{-3}$		−34.40
10 × 90		831.08		−9.75	5.50 × $10^{-3}$		−22.18
1600	1.3	751.44	751.44	0.00	6.07 × $10^{-3}$	6.10 × $10^{-3}$	0.47
32 × 50		763.72		−1.61	6.62 × $10^{-3}$		−7.82
20 × 80		802.82		−6.40	9.79 × $10^{-3}$		−37.65
2500	1.4	791.77	791.77	0.00	7.94 × $10^{-3}$	7.91 × $10^{-3}$	−0.39
40 × 62.5		804.92		−1.63	9.12 × $10^{-3}$		−13.30

**Table 6:** The predictions and errors of the ANN model with a single input volume.

Volume	First-Order Natural Frequency/Hz		Error/%	Displacement Amplitude/mm		Error/%
	COMSOL	ANN		COMSOL	ANN	
405.00	764.52	764.52	0.00	0.70 × $10^{-3}$	2.89 × $10^{-3}$	310.74
506.25	786.92	786.92	0.00	3.33 × $10^{-3}$	2.60 × $10^{-3}$	−22.14
2531.25	700.76	674.24	−3.78	3.07 × $10^{-3}$	10.73 × $10^{-3}$	249.79
2890.00	736.39	736.39	0.00	4.63 × $10^{-3}$	7.41 × $10^{-3}$	59.82
3307.50	813.20	813.20	0.00	11.63 × $10^{-3}$	14.74 × $10^{-3}$	26.74
4000.00	780.70	780.70	0.00	6.13 × $10^{-3}$	4.81 × $10^{-3}$	−21.48
7290.00	953.00	953.00	0.00	12.41 × $10^{-3}$	12.42 × $10^{-3}$	0.09
10,890.00	1120.99	1341.22	19.65	23.11 × $10^{-3}$	27.42 × $10^{-3}$	18.66
13,537.50	1161.84	1161.84	0.00	22.07 × $10^{-3}$	23.27 × $10^{-3}$	5.43

## Data Availability

The data presented in this study are available in this article.
